# Determination and Quantification of the Vinblastine Content in Purple, Red, and White *Catharanthus Roseus* Leaves Using RP-HPLC Method

**DOI:** 10.15171/apb.2018.019

**Published:** 2018-03-18

**Authors:** Rohanizah Abdul Rahim, Nor Hazwani Ahmad, Khaldun Mohammad Al Azzam, Ishak Mat

**Affiliations:** ^1^Advanced Medical & Dental Institute, Universiti Sains Malaysia, 13200 Kepala Batas, Pulau Pinang, Malaysia.; ^2^Preparatory Year Department, Al-Ghad International Colleges for Applied Medical Sciences, Riyadh, Kingdom of Saudi Arabia.; ^3^Department of Chemistry, Dalhousie University, Halifax, Nova Scotia, Canada.; ^4^Unit Kanser MAKNA-USM, Advanced Medical & Dental Institute, Universiti Sains Malaysia, 13200 Kepala Batas, Pulau Pinang, Malaysia.

**Keywords:** Catharanthus roseus, HPLC, Alkaloid, Vinblastine, Plant

## Abstract

***Purpose:*** To determine and quantify vinblastine in different varieties of Catharanthus roseus using reversed-phase HPLC method.

***Methods:*** The liquid chromatographic separation was performed using a reversed phase C18, Microsorb - MV column (250 mm x 4.6 mm, 5 µm) at room temperature and eluted with a mobile phase containing methanol – phosphate buffer (5 mM, pH 6.0) – acetonitrile with different proportion gradient elution at a flow rate of 2.0 mL min^-1^ and detection at 254 nm.

***Results:*** The HPLC method was utilized for the quantification of vinblastine in purple, red and white varieties of Catharanthus roseus leaves. The separation was achieved in less than 8 min. The peak confirmation was done based on the retention times and UV spectra of the reference substance. The method was validated with respect to linearity, precision, recovery, limit of detection and quantification. Results showed that the purple variety gives 1.2 and 1.5 times more vinblastine concentration compared to the white and pink varieties, respectively.

***Conclusion:*** The obtained results from different varieties are thus useful for the purpose of vinblastine production from Catharanthus roseus plant.

## Introduction

*Catharanthus roseus,* commonly known as Madagascar periwinkle which belongs to the Apocynaceae family. It grows naturally as a wild flower throughout Africa, America, Australia, Asia, Southern Europe and on some islands in the Pacific Ocean. The *Catharanthus roseus* is widely studied and used as plant due to its pharmaceutical value that comes from its diversity of useful terpenoid indole alkaloids. The most valuable bisindole alkaloids are vincristine and vinblastine due to their antineoplastic activity in the treatment of many types of cancers.^1^

The difference in color of its flowers indicates the varieties of the plants along with its different biological characteristics.^2^ Aqueous extracts of *Catharanthus roseus* have also been used in traditional medicine for preventing some diseases such as bleeding problems, diabetes, fever, malaria, stomach problems, heart disease and cancer.^3^

**Figure 1:**
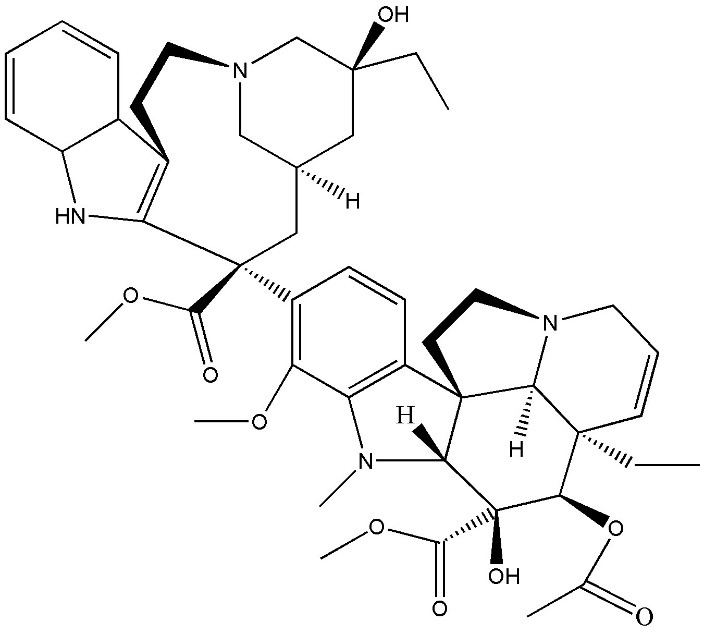
Chemical structure of vinblastine .

The most two important alkaloids present in *Catharanthus roseus* as antitumor compounds are vinblastine (Figure 1) and vincristine which are a derivative of dimerization of vindoline and catharanthine in the vacuole of *Catharanthus roseus* leaves and stem cells.^4^

The conventional methods for extracting traditional herbal medicine products include soxhlet extraction, ultrasonication and reflux. These extraction methods have some drawbacks such as the prolonged preparation time and low efficiency.^5^

Several studies have been conducted to identify these alkaloids using various methods such as high performance liquid chromatography (HPLC) either equipped with ultraviolet (UV)^6^ or photodiode array (PDA) detector,^7,8^ thin layer chromatography (TLC),^9^ and capillary electrophoresis.^10^

To the best of our knowledge, there is no study reported on the vinblastine quantification in *Catharanthus roseus* with different varieties. In the present study, we investigated and compared the vinblastine content in the leaves from three wild local varieties of *Catharanthus roseus* which have purple, pink and white flowers. A simple and fast HPLC method was also developed and validated for the separation and determination of vinblastine in the aqueous extract.

## Materials and Methods

### Chemicals and reagents

All chemicals and solvents used were of analytical and chromatographic grade, respectively. Nanopure water (resistivity, 18.2 M Ω cm^−1^) (Mili-Q18 Barnstead Sterling Ascent, USA) was used for the preparation of samples. Disodium hydrogen orthophosphate dihydrate, orthophosphoric acid (85%), HPLC grade of acetonitrile and methanol were purchased from Fisher Scientific (Darmstadt, Germany). Standard of vinblastine (99%) was purchased from Tocris (Northpoint Fourth Way, UK).

### Preparation of standard solutions

Vinblastine stock solution was prepared at 100 µg mL^-1^ concentration by dissolving the vinblastine standard in the purified water in a universal bottle and stored at -20 ºC. Standard of working solutions were prepared by diluting the stock solution in purified water at 56, 52, 48, 44, 40 and 32 µg mL^-1^ concentrations and filtered through 0.2 µm membrane cellulose filters (Advantec, Japan) for further analysis.

### Samples

Green leaves of *Catharanthus roseus* of purple, pink and white flower were obtained from Melaka, Malaysia.

### Extraction procedure

**Figure 2:**
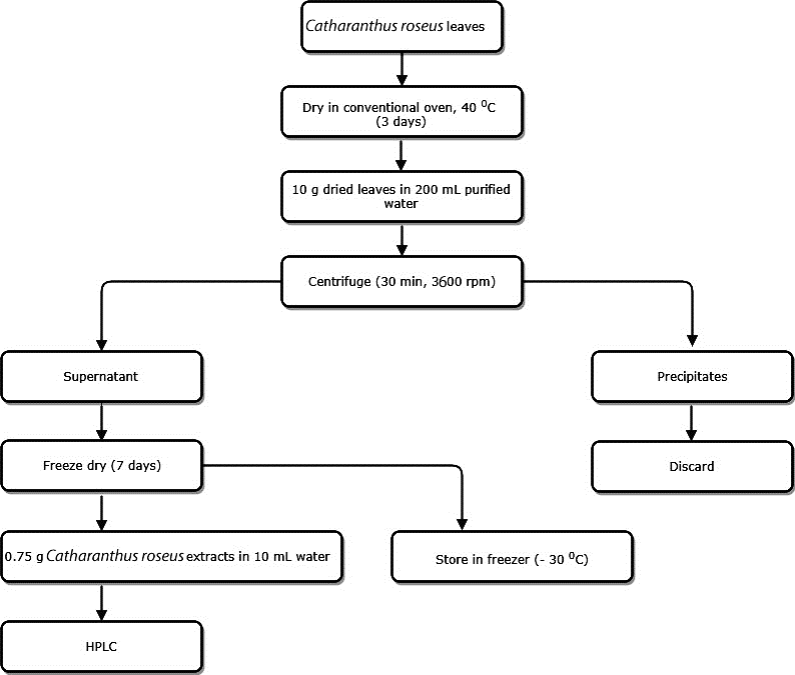
Schematic diagram for the extraction procedure of vinblastine from *Catharanthus roseus* plant.

The leaves were dried using conventional oven (Sharp, Malaysia) at 40 °C for four days. 10 g of the dried leaves were placed in 200 mL water at 40 °C for 24 h in a covered container and placed in the shaker water bath (Memmert, Germany) to obtain the extracts. Once the dried leaves have been disintegrated, the extracts were then centrifuged at 3600 rpm for 30 min in order to yield the supernatants. The supernatants were then freeze dried in order to obtain the powder which was later kept in a refrigerator at -30°C (Sanyo, Japan) for further analysis. The procedure is summarized in Figure 2.

### HPLC analysis and chromatographic conditions

#### Preparation of samples

1 g of aqueous extract was dissolved in 10 mL purified water and filtered through 0.2 µm membrane filters to obtain the sample stock solution and then, diluted to the 75 mg mL^-1^ for HPLC analysis.

#### Identification and quantification of vinblastine in the leaf samples

**Figure 3:**
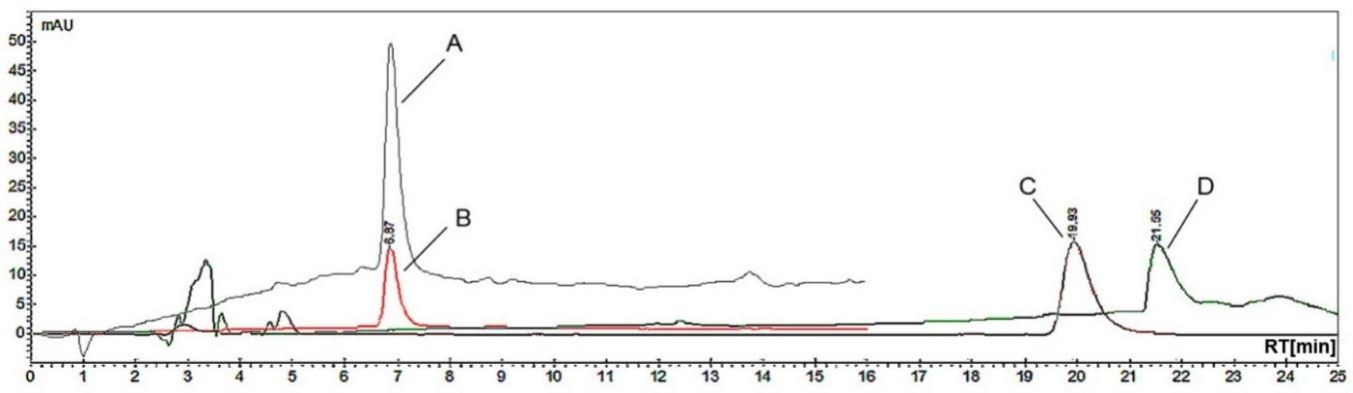
Typical HPLC chromatograms of vinblastine standard at (40 µg mL^-1^). A) 220 nm B) 254 nm using isocratic elution (acetonitrile and phosphate buffer); C) Gradient elution (methanol, acetonitrile and ammonium acetate buffer); D) Gradient elution (acetonitrile and phosphate buffer).

HPLC System (Varians, USA) equipped with a Pro Star solvent delivery system (model 240), photodiode array detector (PDA) (model 335), rheodyne injector with a 20 µL sample loop (model 410) and the Galaxie LS WS software was used. The separation was achieved by using Microsorb-MV C18 reversed phase column (150 mm x 4.6 mm lengths, 5 µm) at room temperature. A gradient mode with the following solvent systems that is A (methanol), B (5 mM phosphate buffer) and C (acetonitrile) was used. The buffer was filtered using nylon 0.2 µm membrane filter (Magna, China) and all solvents were degassed in a sonicator (Power Sonic 405, Korea) for 15 min prior to use. The flow rate was set at 2.0 mL min^-1^. The elution profile starting from 25% A, 40% B, 35% C for 0 min; 35% A, 3% B, 30% C for 5 min; 30% A, 35% B, 35% C at 10 min and 30% A, 35% B, 35% at 15 min. Then the system was allowed to stabilize for 5 min under the initial conditions. The UV detection was conducted at 254 nm instead of 220 nm because it provides a better baseline (Figure 3(A & B)).

## Results and Discussion

Experimental data were analyzed using *t*-test (SPSS Version 13.0, USA). Throughout the analysis, F-values (*P* < 0.05) were considered significant.

### Sample preparation

The leaves were dried before the extraction procedure to ensure the total removal of water from the leaves. The percentage of water in purple, pink and white were 75.8, 85.5 and 85.6%, respectively. The dried leaves were ground until homogenous to ensure that it can improve the kinetic of the analytes in the extraction process.^5^ The extraction method as described by Ferreres *et al*. ^3^ and Tickhomiroff and Jolicoeur^11^ was chosen as it involved the same matrix plant. Slight modification was done to the previous method where in the present study, we used temperature at 40°C and non-toxic solvent (water) instead of boiling water and toxic alcoholic solvent (methanol).^3^ In this study, aqueous extracts is preferable and more importantly, it is safe to be used as a cure or disease prevention in the field of medicine.

The extraction yield of vinblastine was 88% when they used single methanol extraction.^11^ However, aqueous extraction method, allows us to retain higher extracts from the leaves as reported in this study, and this procedure has succeeded to yield about 95 – 103% of vinblastine extracts. The samples were frozen, dried after the extraction procedure in order to reduce the volume of aqueous used without denaturing the alkaloids.^6^

### Method optimization and development of HPLC conditions

The HPLC method was optimized using different proportions of mobile phase such as acetonirile, methanol, sodium phosphate and ammonium acetate buffer. A previous study conducted by Singh *et al*.^12^ showed that the separation of alkaloid can be achieved using isocratic elution. Therefore, initially the most straight forward isocratic elution conditions have been investigated using acetonitrile - phosphate buffer (pH 6.0; 5 mM) (1:1, v/v) at a flow rate of 2.0 mL min^-1^. However, the isocratic conditions showed that there was a peak tailing to vinblastine peak.

Other studies done by Tickhomiroff and Jolicoeur ^11^ used phosphate buffer and Choi *et al*.^13^used ammonium acetate showed that the separation of alkaloids also can be improved by using gradient elution. Therefore, the gradient elution mode was further investigated. The elution process involved the use of A (acetonitrile) and B (5 mM sodium phosphate buffer) at a flow rate of 2.0 mL min^-1^. The mobile phase started with 20% A at 0 min, 80% A at 20 min, 20% A at 25 min and 80% A at 40 min. It was found that the detection of vinblastine standard takes a longer time, which is around 21.55 min (Figure 3(D)) than in the previous study.^11,13^ Therefore, ammonium acetate buffer with acetonitrile has also been tried, but it gave late elution of vinblastine which was around 19.93 min (Figure 3(C)).

The determination and separation of vinblastine were done using gradient elution in three mobile phases consisted of 5 mM sodium phosphate buffer with pH 6.6 and organic solvents of acetonitrile and methanol. Phosphate buffer was used in this analysis to minimize the peak tailing. Results showed that vinblastine elutes faster, which is around 6.87 min (Figure 3 (B)) compared to the previous methods which are around 15.2 min^11^ and 21 min.^13^

### Linearity, limit of detection and limit of quantification

Good linear regression (r^2^ > 0.999) was achieved by injecting triplicate of six concentrations of vinblastine standard (32 - 56 µg mL^-1^) into the HPLC system.

The limit of detection (LOD) and limit of quantification (LOQ) were calculated based on the formula of Shabir:^14^ LOD = 3.3 (SD/S), while LOQ = 10 (SD/S), whereby SD is the standard deviation of response and *S* is the slope in the calibration curve. The value of LOD was 0.0230 µg mL^-1^, while LOQ 0.0698 µg mL^-1^.

### Precision

Repeatability and reproducibility of the method were determined by injecting six times each of the standard solutions (36, 44 and 52 µg mL^-1^) on the same day (intra-day) and over six days (inter-day), respectively. The results showed good repeatability of both the peak area (% RSD, 1.11 - 2.37%) and retention times (% RSD, 0.41 - 3.02%) as shown in Table 1. Student *t*-test was done and the results are statistically significant (Table 1).

**Table 1:** Intra and inter-day precision for the determination of vinblastine.

**Amount (µg mL**^-1^)	**RSD (%) (Area)**	**RSD (%) (Retention time)**
Intra-day precision (n=9)	-	-
36	1.11	0.74
44	2.21	0.41
52	1.19	1.12
Inter-day precision (n = 27)	-	-
36	1.14	1.59
44	1.33	2.22
52	2.37	3.02

Key to abbreviation; RSD: Relative Standard Deviation

### Recovery

Recovery studies were carried out by spiking three concentrations (36, 44 and 52 µg mL^-1^) of vinblastine standard to the samples. The mixtures were extracted and the supernatants were freeze-dried. The obtained aqueous extracts were dissolved in water and injected into the HPLC column. Recovery for each sample was varied from 95 to 103% (CV, 0.5234 - 2.4498%), 95 to 99% (CV, 3.0316 to 4.0754%) and 96 to 101% (RSD, 0.05774 - 0.7069%) for purple, pink and white variety, respectively. The results for recovery are statistically significant as shown in Table 2.

**Table 2:** Accuracy results for the determination of varieties of *Catharanthus roseus* which have purple, pink and white flowers.

**Concentration Level** (μg mL^-1^)	% Recovery*		
	**(Purple Flower)** (Mean ± SD)	**(White Flower)** (Mean ± SD)	**(Pink Flower)** (Mean ± SD)
36	103.0 ± 2.71	98.0 ± 2.71	101.0 ± 3.14
44	95.0 ± 1.28	95.0 ± 2.22	98.0 ± 1.28
52	100.0 ± 2.87	99.0 ± 1.08	96.0 ± 1.28

*Indicates mean of six determinations (n=6); SD: Standard deviation.

### Analysis of sample

**Figure 4:**
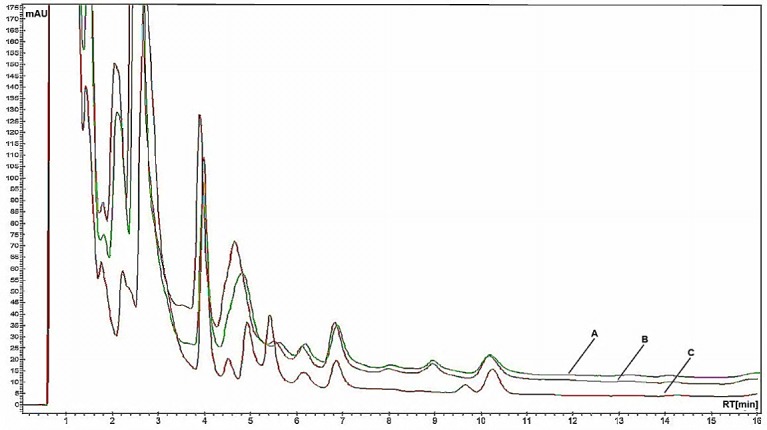
Typical HPLC chromatograms of (A) Purple variety (B) White variety (C) Pink variety.

The peak identification of vinblastine was based on the comparison between retention times and UV spectra of vinblastine standard. We further confirmed the identification by spiking standards to the samples. Vinblastine standard was detected at the retention time around 6.80 min at wavelength 254 nm (Figure 3(B)). The method was applied for the determination of vinblastine in the leaves of three local varieties of *Catharanthus roseus.* The highest concentration of vinblastine was found in purple variety with (0.7320 mg in 1 g) ( Figure 4(A)) compared to the white variety (0.5890 mg in 1 g) (Figure 4(B)) and pink variety (0.4920 mg in 1 g) (Figure 4(C)).

Our work provides a simple extraction method with a simple and rapid HPLC analytical method using a PDA detector. Moreover, the HPLC method also was proposed because it can be scaled-up to the preparative HPLC in the next study and used to isolate pure compounds for biological assays.

## Conclusion

This study proved the existence of vinblastine in *Catharanthus roseus* leaves using reversed-phase HPLC method. Purple variety has 1.2 times and 1.5 times more vinblastine concentrations as compared to the white variety and pink variety, respectively. A simple extraction procedure had effectively yielded vinblastine after separation in crude *Catharanthus roseus* aqueous extracts.

## Acknowledgments

This work has been supported by the Majlis Kanser Nasional (MAKNA).

## Ethical Issues

Not applicable

## Conflict of Interest

All authors declare that she has no conflict of interest.
